# Supplementary material for: Clinical Phenotypes of Progressive Supranuclear Palsy—The Differences in Interleukin Patterns
Source: Int J Mol Sci. 2023 Oct 13;24(20):15135. doi: 10.3390/ijms242015135 (PMC10606588; doi:10.3390/ijms242015135)
Supplement: Supplementary file 1 [file ijms-24-15135-s001.zip › ijms-2647865-supplementary.pdf]

Table S1. Detailed results of blood analysis in patients with PSP.

Initials	Diagnosis	Neutrophils [×10³]	Platelets [×10³]	Lymphocytes [×10³]	NLR	PLR
**SS**	PSP-RS	4.25	194	2.36	1.801	82.203
**SH**	PSP-RS	3.37	147	1.62	2.080	90.741
**PN**	PSP-RS	1.58	163	1.47	1.075	110.884
**PM**	PSP-RS	3.89	261	2.99	1.301	87.291
**MD**	PSP-RS	4.42	264	1.68	2.631	157.143
**ŚT**	PSP-RS	3.51	156	1.19	2.950	131.092
**JZ**	PSP-RS	3.1	175	1.8	1.722	97.222
**WK**	PSP-RS	5.49	282	2.53	2.170	111.462
**KT**	PSP-RS	6.55	280	2.98	2.198	93.960
**BK**	PSP-RS	5.94	205	1.22	4.869	168.033
**RM**	PSP-RS	4.89	223	1.77	2.763	125.989
**GE**	PSP-RS	6.21	172	1.88	3.303	91.489
**RS**	PSP-P	6.88	210	2.81	2.448	74.733
**KA**	PSP-P	4.46	231	2.79	1.599	82.796
**NJ**	PSP-P	5.08	260	1.32	3.848	196.970
**GA**	PSP-P	2.25	119	1.09	2.064	109.174
**GG**	PSP-P	4.79	139	1.57	3.051	88.535
**MT**	PSP-P	3.83	336	2.39	1.603	140.586
**FB**	PSP-P	5.74	266	2.56	2.242	103.906
**MM**	PSP-P	3.82	280	2.66	1.436	105.263
**GA**	PSP-P	6.43	372	3.28	1.960	113.415
**KG**	PSP-P	3.74	219	2.12	1.764	103.302
**LR**	PSP-P	4.77	110	2.54	1.878	43.307
**DJ**	PSP-P	3.96	242	1.23	3.220	196.748
